# MATES in Manufacturing: A Cluster RCT Evaluation of a Workplace Suicide Prevention Program

**DOI:** 10.1002/ajim.23698

**Published:** 2025-01-12

**Authors:** Anthony D. LaMontagne, Christopher Lockwood, Andrew Mackinnon, David Henry, Laura Cox, Neil R. Hall, Tania L. King

**Affiliations:** ^1^ Institute for Health Transformation Deakin University Geelong Australia; ^2^ Centre for Health Equity, Melbourne School of Global and Population Health University of Melbourne Victoria Australia; ^3^ MATES in Construction University of Melbourne Melbourne Victoria Australia; ^4^ Centre for Mental Health & Community Wellbeing, Melbourne School of Global and Population Health University of Melbourne Victoria Australia; ^5^ Australian Manufacturing Workers Union (AMWU) Granville New South Wales Australia; ^6^ Formerly employed at MATES in Construction Melbourne Victoria Australia; ^7^ Centre for Male Health, School of Social Sciences Western Sydney University Penrith New South Wales Australia

**Keywords:** cluster randomized, effectiveness, evaluation, experimental, implementation, intervention, manufacturing, prevention, suicide, trial, workplace

## Abstract

**Background:**

The MATES in Construction suicide prevention program was adapted to the manufacturing sector and evaluated in a pilot of the program.

**Methods:**

Ten manufacturing worksites were randomly assigned to intervention (5 sites) and wait‐list control (5 sites) conditions in a two‐arm cluster randomized design. 1245 workers responded at baseline (87% response rate) and 648 at final (35% response rate). Literacy of Suicide Scale (LOSS) was assessed as a process outcome, and help‐seeking intentions as the primary outcome (General Help‐Seeking Questionnaire [GHSQ] score). Secondary outcomes included help sought, suicidal thoughts and likelihood of suicide attempt scores, and Kessler‐6 scores. Linear mixed models for repeated measures were used in intention‐to‐treat (ITT) and completer analyses.

**Results:**

All sites finished the trial, with intervention periods ranging from 8 to 11 months; however, none of the five intervention sites fully implemented the intervention as planned. ITT analyses showed an improvement in LOSS scores within the intervention group (0.49, 95% CI 0.13–0.49), but the mean difference in change between intervention and control included the null (0.34, 95% CI −0.10 to 0.80). The primary outcome of GHSQ scores also improved within the intervention group, but the difference in change included the null (mean difference 1.52, 95% CI −0.69 to 3.74). No secondary outcomes improved relative to control in ITT or completers analyses. Exploratory analysis of disaggregated GHSQ help sources showed greater improvement in mean difference in change for the main MATES message of seeking help from MATES Connectors.

**Conclusion:**

The intervention, as implemented, was not effective at achieving the primary or secondary outcomes.

**Trial Registration:**

Australian and New Zealand Clinical Trial Registry: ACTRN 12622000122752.

## 1 Introduction

Suicide and suicidal behaviors are a critical public health issue globally [1]. More than 700,000 people die by suicide each year worldwide, and the world is not on track to reach the WHO 2030 target of reducing the global rate of suicide by one‐third [2, 3, 4]. Suicide rates are generally 3−4 times higher for men than for women in developed countries [5], and for every death by suicide, an estimated that 10−20 individuals attempt suicide and 17% of all suicide attempts cause permanent disability [6]. This highlights the importance of suicide prevention efforts [3], including in workplace settings [6].

Previous research has shown that suicide rates in predominantly male, blue‐collar work contexts, such as in the building and construction sector, are elevated compared to other workers [7]. In response, targeted workplace suicide prevention efforts have been implemented in construction and some other contexts, such as mining and energy. *MATES in Construction* is one such program (https://mates.org.au/). The MATES in Construction program was established by and for the Australian construction industry in 2007 [8]. It has also been adapted for the mining and energy sectors, and evaluation studies in all of these sectors have demonstrated the program's positive impact on participants' suicide prevention literacy and willingness to seek and offer help [8, 9, 10, 11, 12]. However, the evidence to date has been limited almost entirely to observational studies [12], with only one randomized trial reported to date [13]. The one randomized trial showed that the MATES in Construction program's effectiveness could be enhanced by addition of a MATES App to complement and reinforce the program's main messages [13]. There remains a need for trial‐based evidence to demonstrate MATES program effectiveness.

Based on a reasonable assumption that the MATES program will be relevant and potentially effective in other predominantly male, blue‐collar industries, the MATES program has been adapted for rollout in the manufacturing sector. The impetus for this came from within the manufacturing industry and was driven by a decision at the Australian Manufacturing Workers' Union (AMWU) National Conference in 2019, following a number of suicides across its membership in the proceeding years. Both employers and unions had identified an unmet need for workers to receive support for suicidal distress and petitioned MATES in Construction to adapt its program for the manufacturing sector. MATES in Construction agreed to oversee the creation and implementation of the MATES in Manufacturing program and to roll out the program in collaboration with a joint labor‐management Steering Group in NSW.

Subsequent to the development of the MATES in Manufacturing initiative, funding was sought and obtained to evaluate the implementation and effectiveness of the program using a cluster‐randomized controlled trial (cRCT) design. This article reports on the quantitative aspects of the implementation evaluation, and the quantitative effectiveness evaluation, as previously described in a published protocol [14]. Predictors of suicide risk were evaluated as outcomes due the ethical and feasibility challenges of evaluating suicidal behavior outcomes. The following Research Questions (RQ) are addressed:

1. Was the MATES in Manufacturing program implemented as designed?
2. Was suicide prevention literacy increased by the MATES in Manufacturing program relative to waitlist controls? (process evaluation).
3. Did intentions to seek help increase in the MATES in Manufacturing program relative to waitlist controls? (primary outcome).
4. Did help‐seeking behavior increase, and levels of distress and suicidal ideation decrease, in the MATES in Manufacturing program relative to waitlist controls? (secondary outcomes).

## 2 Methods

### 2.1 Evaluation Study Design

The MATES in Manufacturing intervention was evaluated using a mixed methods implementation evaluation combined with a two‐arm cluster‐randomized trial design with wait‐list controls for effectiveness evaluation. Implementation was evaluated quantitatively by monitoring numbers and types of intervention activities and associated participation rates as well as suicide prevention literacy as a process evaluation measure. Qualitative implementation evaluation will entail characterizing barriers and facilitators to implementation as well as participant experiences [14]; these results will be reported separately.

The trial was registered with the Australian and New Zealand Clinical Trial Registry (ACTRN 12622000122752, 25 January 2022), and details of the study were published in a protocol paper in accordance with SPIRT Guidelines [14]. The design, conduct, and reporting of this trial adhered to the Consolidated Standards of Reporting Trials (CONSORT) guidelines. Following SPIRIT Guidelines, the cRCT evaluation assessed help‐seeking intentions as the primary outcomes, and help sought, suicidality, and psychological distress as secondary outcomes. The protocol was reviewed and approved by the Deakin University Human Research Ethics Committee (protocol #2021‐276).

Deviations from the protocol as initially registered were due to disruptions caused by COVID‐19 restrictions as well as industrial actions at some of the sites. We originally planned for data collection by survey at three time points: baseline (pre‐allocation), 6 months from the start of intervention, and 12 months from start of intervention. The effectiveness evaluation analysis for primary and secondary outcomes relies only on comparing intervention to waitlist control sites over the first two time points. The additional time point at 12 months was intended to assess the sustainability or continuing improvement of any changes observed after the first 6 months of intervention. Because the waitlist control sites started the intervention at time 2, this would have been a descriptive, uncontrolled assessment of change up to 12 months within the intervention arm only. Due to difficulties with recruitment and intervention implementation, we obtained Ethics amendments to drop the 3rd timepoint of data collection and shift timepoint 2 from 6 to 8 months, thereby allowing more time for implementation and to minimize burden on participating sites and workers.

There were also challenges in obtaining paid work time for in‐person administration of follow‐up (time point 2) surveys. Notably, some sites refused to provide this time, and Ethics agreements were amended to allow self‐administration of follow‐up surveys. Finally, following the difficulties above, and the fact that the survey for our economic evaluation was a supplement to the follow‐up surveys, the planned economic evaluation [14] was compromised by very low response rates.

### 2.2 Intervention Description

MATES in Manufacturing is an adaptation of the MATES in Construction workplace suicide prevention program (https://mates.org.au/). Details of the intervention were provided in the published protocol paper [14].

**Figure 1:**
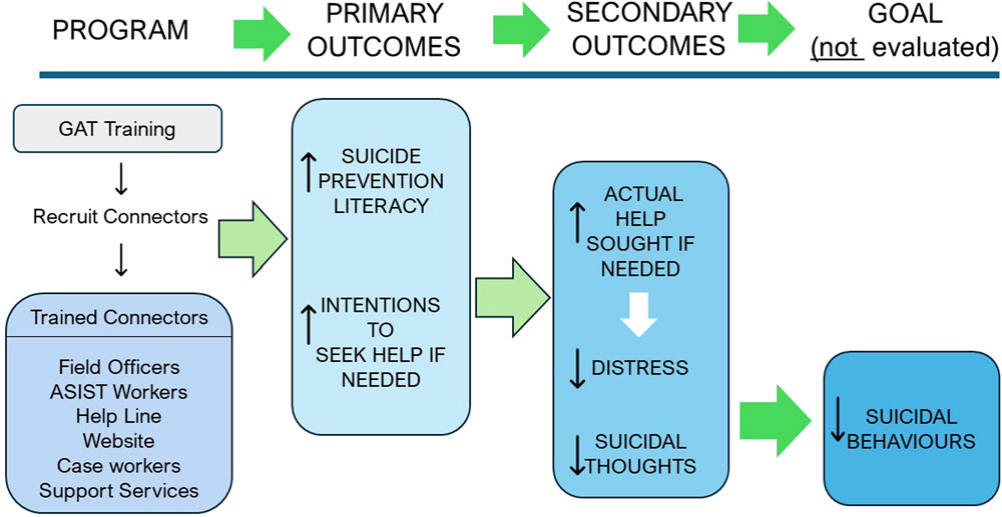
MATES in Manufacturing distilled program logic.

In brief, MATES is an industry‐based community development approach to workplace mental health and suicide prevention. MATES does not provide clinical services but seeks to connect distressed workers to a range of clinical and nonclinical supports. Initially, a 1‐h universal General Awareness Training (GAT) is provided for all workers on a site. Volunteers for half‐day “Connector” training are recruited during the GAT, and subsequently trained as the persons on site who can “connect” workers in need to appropriate sources of help. Distressed workers can either approach a Connector themselves, or a Connector might approach someone based on observation, or concerns expressed by a workmate. “Help” in this context is for mental health or suicidality, or for any issues that could contribute to distress, such as financial stress, alcohol and drug use, or relationship concerns. Volunteers are fundamental to the implementation of the MATES program, and the program aims to have a minimum of 1 in 20 workers (5%) on‐site trained as volunteer Connectors. In addition, MATES also recruits volunteers for Applied Suicide Intervention Skills Training (ASIST): a 2‐day training that equips participants with the skills to codevelop a safety plan with an individual at immediate risk of suicide. The program aims to have at least one or two ASIST‐trained workers on each worksite. On‐site Connector and ASIST volunteers and MATES Field Officers are complemented by a toll‐free MATES help‐line, the MATES website, and case workers. Figure 1 presents a simplified program logic.

Implementation of GAT was the starting point of the intervention at each sites. For those sites randomly assigned to intervention, the intervention was planned to occur for 8 months, and then continued beyond that time point at the discretion of the participating sites. Similarly, for sites randomly assigned to the wait‐list control condition, the intervention was planned to begin 8 months from the date of baseline assessment. The MATES in Manufacturing intervention was implemented by the MATES in Construction NSW organization.

### 2.3 Study Setting and Participants

#### 2.3.1 Recruitment of Study Sites (Clusters)

Sites were recruited through the MATES in Manufacturing Steering Group, which included representatives from interested manufacturing companies, the Australian Industry Group (AIG), unions representing workers at interested sites (Australian Manufacturing Workers' Union, and Australian Workers' Union), representatives from the MATES in Construction National organization (C.L., L.C., R.B.), and the lead investigator of the trial (A.D.L.). Site inclusion criteria were: must be a manufacturing company located in the state of New South Wales, and preferably employing at least 100 workers.

Site recruitment commenced in August 2020 but was hampered by the COVID‐19 pandemic and the associated restrictions and impacts on businesses resulting in recruitment not being completed until December 2021 when the pilot was officially launched. As sites were recruited, they were grouped and randomly assigned as soon as feasible, such that data collection and intervention implementation could be spread out over time and managed with the small implementation and research teams available. Two groups of four sites, and one final group of two sites, were recruited and baseline data collection completed before random assignment.

Recruitment of Participants within Sites: All workers employed at each participating site were eligible for the MATES program, and all were invited to participate in baseline and follow‐up surveys (census sampling). There were three criteria for inclusion, participants must (1) be employees or workers in the manufacturing industry and working at MATES in Manufacturing participating sites (this included casual/temporary, contract, and continuing/permanent employees); (2) have basic English, Vietnamese, or Mandarin proficiency. While program activities were conducted in English, surveys and some printed information on the program were also translated into Vietnamese and Mandarin, and made available on an as‐needed basis (reflecting the high Vietnamese and Chinese composition of some sites); and (3) be at least 18 years old.

### 2.4 Sequence Generation, Randomization, and Allocation Concealment

Allocation of sites (clusters) to intervention arms was conducted by the project statistician (AM) using minimization implemented with the *rct_minim* procedure in Stata. Balance was sought for a single factor – site size – with sites of fewer than 150 workers classified as “small” and sites of 150 or more workers classed as “large.” Due to resource limitations, participating sites were randomly assigned to intervention or wait‐list control in small batches of up to four sites (further details below). Allocations were then revealed to the research team, MATES field staff and sites, as blinding beyond the baseline assessments was not feasible.

### 2.5 Data Collection: Quantitative Effectiveness Evaluation

Survey data was collected by MATES Field Officers and research staff. Data collection at each worksite was agreed a priori to occur within work hours, at dates and times agreed with the company/site managers [14]. For baseline surveys, MATES staff delivered a brief preamble about the study purpose and aims, and informed workers that workers that program participation was not contingent upon completion of the (voluntary) surveys. The Informed Consent process, Plain Language Statements, Consent Forms, and safety procedures were detailed in the published protocol [14].

### 2.6 Measures

The hypothesized relationships between program activities, process outcomes, primary outcomes, secondary outcomes, and future risk of suicidal behavior are depicted in Figure 1.

#### 2.6.1 Quantitative Implementation Evaluation

We monitored and recorded the number of program activities and participation levels in program activities (e.g., percentage of workers on site attending GAT; number and percentage of GAT trainees recruited and successfully trained as Connectors). In addition, we measured suicide prevention literacy as a process/implementation measure using the Literacy of Suicide Scale Short form (LOSS) [15]. Each of the 12 items on the LOSS is responded to on a 3‐point scale (“True”, “False”, or “I don't know”), with correct responses allocated a score of 1 and incorrect or “I don't know” responses assigned a score of 0. Total scale scores were calculated by summing item scores, yielding a total literacy score with higher scores indicating higher literacy. The LOSS has been validated by using Item Response Theory to identify items that had the strongest discrimination of the underlying literacy construct [15].

#### 2.6.2 Help‐Seeking Sources and Intentions (Primary Outcome)

Help‐seeking intentions were measured using the General Help‐Seeking Behavior Questionnaire (GHSQ) [16]. GSHQ questions were modified to present twelve distinct sources of help (including a MATES worker or Connector and an open‐ended option for ‘other’), and participants were asked to rank their help‐seeking intentions prefaced by: “if you were feeling overwhelmed and unable to cope.” This phrasing was derived from a qualitative study of how blue‐collar male workers conceptualize emotional and suicidal distress, with the results indicating that blue‐collar male workers are inclined to emphasize a loss of agency [17]. Intentions were ranked from *extremely unlikely* to *extremely likely on a 7‐point scale* [16]. A single summary measure across all the 12 options was computed by summing the responses (reverse scoring the “no one” option). The higher the score, the greater the help‐seeking intentions.

#### 2.6.3 Help Sought (Secondary Outcome)

Among those who reported having felt overwhelmed or unable to cope at any point in the preceding 6 months (yes/no), forms of help sought during the preceding 8 months were measured using the GHSQ [16]. GSHQ questions listed the same 12 sources of help as detailed in the primary outcome of help‐seeking intentions, except in this case asking whether help has been sought, and how frequently (*never/rarely/sometimes/often/always*). A single summary measure across all the 12 options was computed by summing the responses (reverse coding the *No one* option). The higher the score, the greater the help sought.

#### 2.6.4 Suicidal Ideation (Secondary Outcome)

Suicidal ideation was measured using one item from the Suicidal Behavior Questionnaire‐Revised (SBQ‐R) [18], assessing the frequency of suicidal ideation in the past 6 months on a scale from *Never* (1) to *Very Often* (5). The higher the score, the more frequent (worse) the suicidal ideation.

#### 2.6.5 Likelihood of Suicide Attempt (Secondary Outcome)

Likelihood of suicide attempt was measured using one item from the Suicidal Behavior Questionnaire‐Revised (SBQ‐R) [18] asking about the likelihood that the respondent *will attempt suicide someday*. Response options were on a six‐point scale from *Never* (0) to *Very Likely* (5). The higher the score the higher (worse) the likelihood of suicide attempt.

#### 2.6.6 Psychological Distress (Secondary Outcome)

Psychological distress was measured using the Kessler‐6 instrument (K6) [19]. Participants were asked to indicate the response that best describes their feelings in the past 4 weeks for each of six items. Responses were on a 5‐point Likert scale ranging from 0 (*none of the time*) to 4 (*all of the time*). The six items were summed to give a score ranging from 0 to 24. The higher the score the greater (worse) the psychological distress.

#### 2.6.7 Covariates

Unique participant identifiers were generated by MATES and provided to researchers for within‐person linking of baseline and final assessments, company/site name (for cluster identification), age, gender, occupation, Aboriginal and Torres Strait Islander status, country of birth (Australian‐born: Yes/No), history of previous training in MATES programs (Yes/No), which may have occurred through previous employment in Construction, Energy, or Mining.

### 2.7 Analysis

#### 2.7.1 Implementation

Descriptive analyses detailed the occurrence and frequency of program intervention activities relative to plans and participation levels in program activities, to address the Research Question: Was the MATES in Manufacturing program implemented as designed?

The suicide prevention literacy process (LOSS) measure collected by survey was analyzed as below with the quantitative effectiveness evaluation to address the Research Question: Was suicide prevention literacy increased by the MATES in Manufacturing program relative to wait list controls?

#### 2.7.2 Effectiveness

The primary analyses were undertaken on an intention‐to‐treat (ITT) basis: including all participants as randomized, regardless of treatment received or withdrawal from the study. Linear mixed models for repeated measures (MMRM) were used because of the ability to include participants with missing data, with factors of group and time and their interaction. Within‐person associations were accommodated using an unstructured variance‐covariance matrix. Potential clustering effects within sites were modeled using a random intercept. The Kenward–Roger method, based on the observed information matrix, was used to adjust degrees of freedom for tests. The statistical significance of each outcome was evaluated in a planned comparison of change from baseline to follow‐up in the active/MATES arm compared to change in the waitlist control group. Primary ITT analyses were complemented by “Completers” analyses of participants who completed both baseline and follow‐up surveys.

In addition, as specified in the protocol [14], we conducted exploratory analyses to investigate whether intervention‐associated changes differed by gender or by manual blue‐collar versus white‐collar occupational status.

#### 2.7.3 Power and Sample Size

We based our power and sample size calculations on the primary outcome of GHSQ intentions to seek help, as detailed previously [14]. Power calculations indicated that we would have adequate power to detect an effect size of 0.3 Cohen's *d* standardized difference in our primary outcome of GHSQ Help‐seeking intentions with 10 sites, each including at least 100 workers, or 14 sites, each including at least 50 workers [14]. The Steering Group tried to recruit at least 12 sites to protect against attrition but was only able to recruit 10 sites, four of which employed fewer than 100 workers. This was in part due to COVID site visitation restrictions at the time, as well as sites determining that requirements of trial participation were too cumbersome.

## 3 Results

### 3.1 Participant Flow and Numbers Analyzed

**Figure 2:**
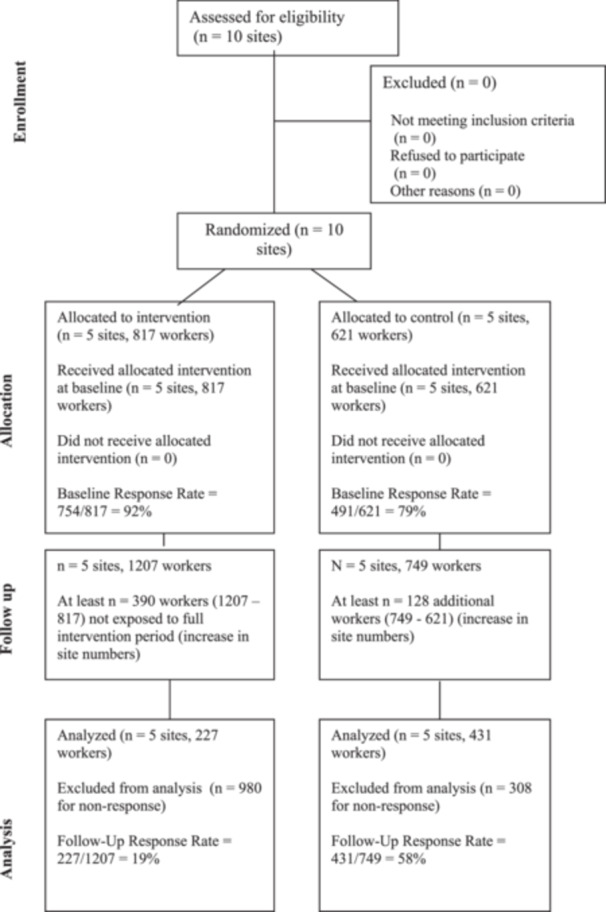
MATES in Manufacturing CONSORT flow chart of participating sites (clusters) and workers (individual participants).

Despite successfully recruiting 10 sites with a total of 1438 workers (greater than the minimum of 1000 workers from 10 sites), four of the sites employed fewer than 100 workers (Table 1). Figure 2 presents the CONSORT flow diagram for sites and individual participants. All 10 sites that started in the trial completed, with no sites dropping out.

**Table 1:** MATES in Manufacturing pre‐randomization baseline and follow‐up survey completion rates.

Site	Condition	Baseline survey		Follow‐up survey	
		Site size (*N*)	Completion rate, *N* (%)	Site size (*N*)	Completion rate, *N* (%)
1	Intervention	25	23 (92)	25	17 (68)
2	Control	70	65 (93)	88	66 (75)
3	Intervention	122	122 (100)	142	67 (47)
4	Control	321	243 (76)	321	151 (47)
5	Intervention	100	89 (89)	150	18 (12)
6	Intervention	350	304 (87)	440	81 (18)
7	Intervention	220	216 (98)	450	44 (10)
8	Control	50	36 (72)	60	41 (68)
9	Control	60	39 (65)	60	39 (65)
10	Control	120	108 (90)	220	134 (61)
	**Total**	**1438**	**1245 (87)**	**1956**	**658 (34)**

Participation in surveys before randomization was excellent, with 6 of 10 sites attaining 90% or higher participation, with the lowest achieving 65% (Table 1). Follow‐up survey participation, however, was far lower. Site compliance with per protocol on‐site survey administration by MATES was good at baseline; however, this required up to five scheduled survey administration sessions on paid work time at one site, which this and other sites found difficult to manage. Following an Ethics amendment, this was supplemented with self‐administration at some sites to maximize participation rates. At follow‐up, all sites included self‐administration of surveys, with little or no work time sessions, due to most sites' inability or refusal to allow follow‐up survey administration on paid work time.

There were also substantial increases in the number of workers on site between baseline and follow‐up. Notably, the three sites with very poor participation rates at follow‐up were all intervention sites (12%, 18%, and 10%) and experienced substantial increases in employee numbers from baseline (Table 1, Sites 5–7). These increases were reportedly due to staff numbers having been reduced in line with stringent COVID restriction (which were in effect during baseline assessments), and then raised as COVID restrictions eased (which coincided with follow‐up). Approximately 500 workers who were approached for follow‐up surveys were not on site at baseline; 390 of these were at intervention sites and would not have been exposed to the full intervention period.

### 3.2 Implementation of MATES in Manufacturing Program

Post‐randomization, the MATES program was launched at the five intervention sites with 1‐h General Awareness Training (GAT) sessions for all workers on‐site. This proved to be logistically challenging due to COVID restrictions (e.g., large group training was precluded unless it could be held outdoors), the need to keep manufacturing production processes running on worksites (leading to a need for multiple small sessions at different times for each shift), and industrial actions at two of the sites. This resulted in substantial delays from baseline assessment to program launch from 2 to 12 months. MATES ran two GAT sessions for the smallest site (*n* = 25 at baseline), but required 16 separate sessions for the largest (*n* = 350 at baseline). Intervention periods (time from launch of GAT training to follow‐up surveys) were longer than the 8 months planned for most sites, and extended by up to 3 months at one site: intervention periods at the five sites were 8, 9, 9, 10, and 11 months.

Despite these challenges, there were signs of high engagement from participating workers. The final question on the baseline surveys states, “if you are going through a difficult time, MATES can offer support,” offering the respondent to tick a box for a call back from MATES staff. Call‐back requests varied from 0 (at the smallest site of 25) up to 24 (~9% of participants at a large site). Further, volunteering for ½‐day Connector training and 2‐day ASIST training was high, ranging from 6/23 GAT participants (26%) at the smallest site to 62/271 at one of the larger sites (23%), greatly exceeding the target of 5% of workers on a site being Connector‐trained.

Implementation of Connector training, however, was delayed and incomplete (not all volunteers were trained) due to the same challenges as outlined above (Table 2). Connector training on most sites was not offered until more than half‐way through the intervention period, whereas it was intended to be implemented soon after GAT training. Thus, Connector training did not occur for many volunteers, and when Connector training was provided it occurred well into the intervention period, thus limiting the opportunity for Connectors to engage with workers and have the opportunity to ‘connect’ workers in need to sources of help. ASIST training was not implemented for any of the volunteers during the intervention period (though it was implemented after follow‐up surveys).

**Table 2:** MATES in Manufacturing implementation summary for intervention sites.

Site	Site size	GAT		Connector		ASIST	
		*N* (%)	Level met?^a^	*N* (%)	Level met?^a^	*N* (%)	Level met?^a^
1	25	19 (76)	No	11 (44)	Yes	0	No
3	132	109 (83)	Yes	22 (17)	Yes	0	No
5	125	83 (66)	No	8 (6)	Yes	0	No
6	395	271 (69)	No	27 (7)	Yes	0	No
7	335	220 (66)	No	48 (14)	Yes	0	No

^a^ Level met refers to a minimum of 80% of on‐site workers GAT trained, a minimum of 1 in 20 trained Connectors to on‐site workers ratio (5%), and access to at least one site ASIST‐trained workers (https://mates.org.au/site-accreditation). Site size for these calculations used the mean of baseline and follow‐up worker numbers, which will overestimate % GAT trained at sites with increasing numbers during the intervention period (only one GAT training was offered at baseline).

MATES program accreditation states that 80% of workers on site should be GAT trained, 1:20 (5%) workers should be Connector trained, and each site should have at least 1 ASIST‐trained worker. By these criteria, only one site achieved 80% GAT training, and none of the five sites attained full accreditation status during the evaluation period (Table 2).

A small number of distressed workers were identified and followed up during survey administration. Completed baseline surveys were promptly reviewed and workers at both intervention and wait‐list control sites who had recorded concerning responses to the question about likelihood of a suicide attempt (*likely* or *very likely*, *n* = 25) where phoned and offered case management support. While strictly speaking, this would constitute “contamination” of wait‐list control sites (*n* = 12 responses of likely suicide attempt from control sites), this is a limitation that is ethically imperative and was considered unlikely to substantially affect the overall results of the trial.

### 3.3 Effectiveness Evaluation

Baseline distributions of covariates and outcomes are detailed in Table 3. Given the cluster randomized design, it was appropriate to test for differences in baseline distributions of key variables at the individual respondent level. Substantial and statistically significant differences were observed in four covariates. A higher proportion of “Country of birth other than Australia” and “previous MATES training” were observed in intervention relative to control sites (Table 3). Further, the percentage reporting feeling overwhelmed or unable to cope in the past 6 months was higher in the intervention sites, which was also reflected in higher Kessler‐6 scores at a marginal level of statistical significance (0.07). In contrast, literacy of suicide scale (LOSS) scores were higher (better) in intervention versus control sites.

**Table 3:** MATES in Manufacturing baseline distribution of covariates and outcomes.

	Control (*n* = 491 persons over 5 sites)	MATES in Manufacturing (*n* = 754 persons over 5 sites)	Test for baseline difference (*p* value)*
Gender (% male vs. female or other)	87.2%	89.6%	0.2138
Age (mean)	48.2 years	43.7 years	0.0910
Occupational group (% blue‐collar vs. white collar)	68.0%	66.6%	0.9604
Aboriginal or Torres Strait Islander descent (indigenous status) (%)	2.5%	3.2%	0.4589
Country of birth (% Australia vs*.* other)	38.6%	76.9%	0.0009
Previous MATES training (% with previous training)			
All types combined GAT (%) Connector (%) ASIST (%)	2.1% 1.0% 0% 0%	4.8% 1.9% 0.5% 0.4%	0.0170 0.2596 — —
Literacy of Suicide Scale (LOSS) score (mean (SD))	5.15 (2.86)	6.41 (3.07)	0.0107
General Help‐Seeking Questionnaire (GHSQ) score (mean (SD))	46.33 (13.29)	45.25 (11.93)	0.1609
Felt overwhelmed or unable to cope in the past 6 months (%) If yes, actual help sought score (mean (SD))	22.9%	33.0%	0.0003
	24.35 (7.86)	23.13 (6.76)	0.1522
Suicidal Behavior Questionnaire (SBQ‐R) score			
Suicidal thoughts (1–5, *Never to Very Often*, mean (SD)) Likelihood of attempt (0–5, *Never to Very Likely*, mean (SD))	1.23 (0.67) 0.45 (0.91)	1.27 (0.72) 0.58 (0.98)	0.6782 0.1585
Kessler‐6 psychological distress scale score (mean (SD))	3.30 (3.62)	4.17 (4.07)	0.0741
Respondents who checked box requesting a follow‐up call (%)	4.1%	2.7%	0.1679

* Test for baseline difference using mixed linear or logistic regression models with random site (cluster) effect.

#### 3.3.1 Process Measure

There was significant improvement from baseline to follow‐up in LOSS scores within the intervention group, but the mean difference in change between intervention and controls included the null (Table 4). It is noteworthy, however, that the standardized mean (SMD) difference was moderate (0.50), and considerably larger than the corresponding SMD values for the primary and secondary outcomes.

**Table 4:** Model‐predicted means and mean differences in outcomes by group and mean difference in change: intention‐to‐treat analyses.

	Wait‐list controls (*n* = 5 sites)			MATES in Manufacturing (*n* = 5 sites)			Final results	
Outcome	Baseline	F‐U	Mean difference (95% CI)	Baseline	F‐U	Mean difference (95% CI)	Mean difference in change (95% CI)	Standardized Mean Difference^a^ (95% CI)
*Primary outcome:*								
General Help‐Seeking Scale (GHSQ) score (higher is better)	45.92	46.12	0.20 (−1.19 to 1.60)	45.15	46.88	1.73 (0.01– 3.45)	1.52 (−0.69 to 3.74)	0.06 (−0.11 to 0.23)
*Secondary outcomes:*								
Actual help sought scale (GHSQ) score (higher is better)	25.22	24.64	−0.58 (−2.31 to 1.14)	23.77	22.66	−1.11 (−3.03 to 0.81)	−0.53 (−3.11 to 2.05)	−0.21 (−0.46 to 0.02)
Suicidal thoughts score (lower is better)	1.25	1.23	−0.02 (−0.09 to 0.06)	1.29	1.26	−0.03 (−0.12 to 0.06)	−0.02 (−0.13 to 0.10)	−0.04 (−0.23 to 0.15)
Likelihood of suicide attempt score (lower is better)	0.49	0.43	−0.06 (−0.16 to 0.03)	0.60	0.51	−0.08 (−0.20 to 0.03)	−0.02 (−0.17 to 0.13)	−0.09 (−0.29 to 0.11)
Kessler‐6 psychological distress scale score (lower is better)	3.64	4.00	0.36 (−0.05 to 0.77)	4.34	4.05	−0.29 (−0.81 to 0.23)	−0.65 (−1.31 to 0.01)	−0.01 (−0.20 to 0.18)
*Process outcome:*								
Literacy of Suicide Scale (LOSS) score (higher is better)	5.13	5.27	0.14 (−0.13 to 0.42)	6.39	6.88	0.49 (0.13–0.85)	0.35 (−0.11 to 0.80)	0.50 (0.27–0.73)

^a^ SMD, standardized mean difference at follow‐up. Positive values indicate a better outcome in MATES compared to waitlist at follow‐up.

#### 3.3.2 Primary Outcome

For the primary outcome of GHSQ help‐seeking intentions, there was an improvement from baseline to final within the intervention group, but the difference in change between intervention and control included the null (Table 4). Cross‐sectionally, differences between groups were not statistically significant on either occasion. Further, the SMD post‐intervention was 0.06, a negligible effect size. A Completers analysis, including only the 287 participants with GHSQ scores on both occasions of measurement, showed an overall pattern of results that were almost identical to the full sample (see Supporting Information).

A priori specified sub‐group analyses [14] of the help‐seeking intention outcome by gender suggested that males were more positively affected by the intervention, but this finding was limited by the small number of female respondents and differences in baseline mean GHSQ scores (see Supporting Information). There was no evidence of differential change by occupational status analyzed as blue‐ versus white‐collar occupations (see Supporting Information).

**Figure 3:**
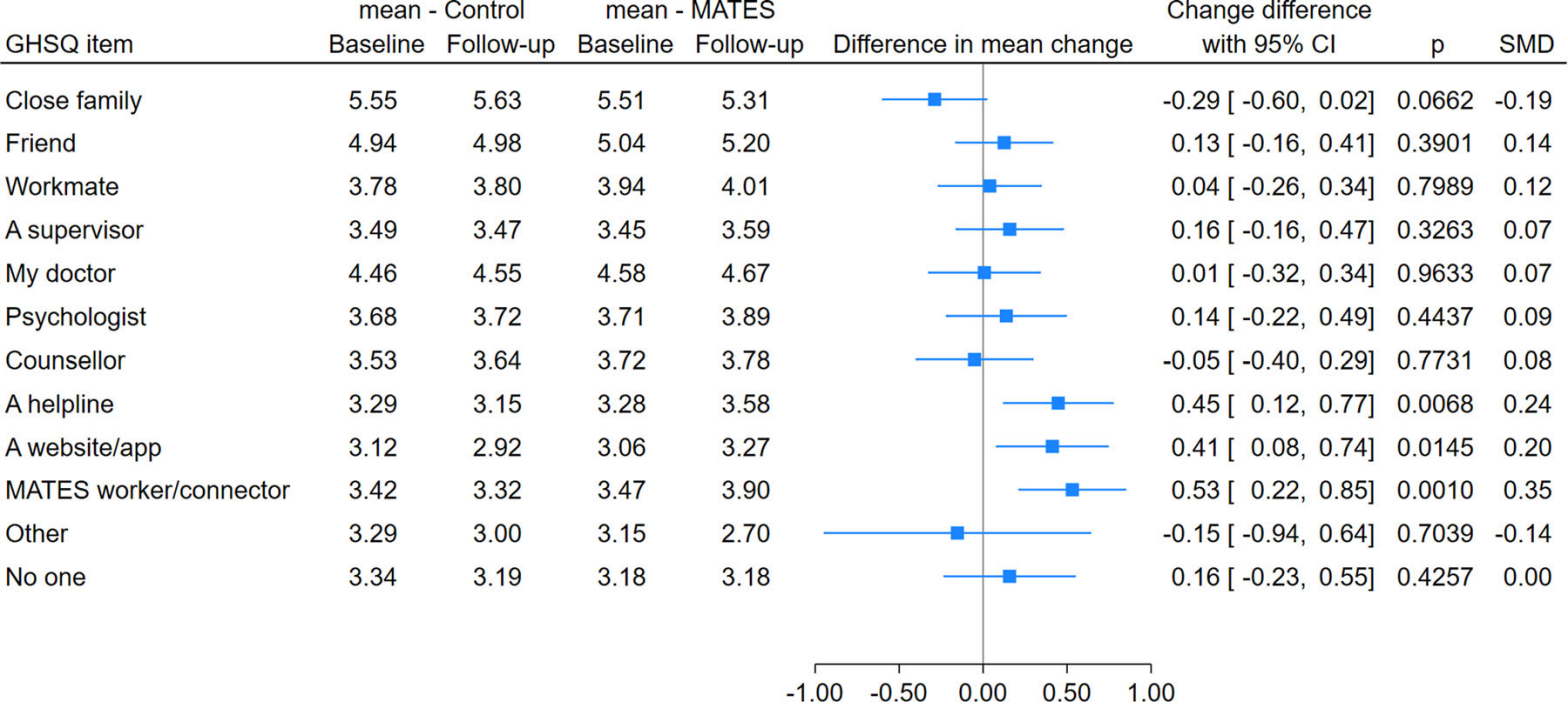
MATES in Manufacturing forest plot of results for disaggregated General Health Seeking Questionnaire (GHSQ) items.

Exploratory analyses were also undertaken by GHSQ items (Figure 3). This analysis showed statistically significant improvements in items corresponding to MATES messaging, which encourages seeking help from a MATES worker or Connector; a website or app; and a helpline. Effect sizes were in the moderate range (0.41–0.53). After further Bonferroni adjustment for multiple testing, only the MATES worker/Connector item showed favorable differential change and excluded the null (data not shown).

#### 3.3.3 Secondary Outcomes

There was no evidence of improvement in intervention versus control sites for any of the 4 secondary outcomes (Table 4). Mean differences in change between intervention and control sites included the null and had small or negligible SMDs (Table 4). Completer analyses of secondary outcomes yielded similar results (data not shown).

## 4 Discussion

This study was conducted to provide an important complement to previously published observational evaluation evidence on the MATES workplace suicide prevention programs. However, the program was only partially implemented, plagued by delays and other challenges related to COVID restrictions, industrial actions, and other factors. Nevertheless, there was some evidence that the program was beginning to work as intended, with a suggestion of improvement in the suicide literacy process measure and evidence of improved mean differences in change in the GHSQ items aligned with key messages of the MATES program. Strictly speaking, however, it was the overall GHSQ help‐seeking intention measure that was our a priori primary outcome, hence we are obliged to conclude that the MATES in Manufacturing intervention was not effective as implemented. Overall, our results suggest that the lack of improvement in the primary and secondary outcomes is mainly due to shortcomings in implementation.

The findings of this report are complemented by a white paper qualitative implementation evaluation of the MATES in Manufacturing trial [20]. Forty workers across five intervention pilot sites volunteered for in‐depth interviews, complemented by four volunteers from the Project steering committee and Mates Field Officers. A number of key enablers and barriers to program implementation were identified, including the importance of having a strong a workplace advocate, key management support, program flexibility, work group‐based communication and peer support, and incidental messaging. Significant barriers included the variable levels of interest and engagement by management and workers, low Connector visibility, latent and enduring stigma, and the challenges of accommodating gender and cultural diversity at participating sites. Overall, the training was rated highly by interview participants, with reports of positive outcomes from the training, including increased mental health literacy and confidence in help‐offering. Post‐training, there were reports of reduced stigma but no discernible difference to date in help‐seeking behaviors, likely as a result of the short time period between training and interviews. Sites witnessed management adopting more empathic and supportive practices, indicating some organizational shift in priorities; however, there was little reference to the systematic development of healthy workplace policy. There was also some reported confusion about the roles of the differing stakeholders in the project (e.g., in terms of who was responsible for organizing and recruiting to the programs) and some frustration about delays in program roll‐out (due to COVID, administrative issues, and industrial actions). Overall, the qualitative component of the research provided a more positive impression of the impacts of the program; however, the self‐selected volunteers for the interview primarily identified themselves as champions of workplace suicide prevention. In summary, the findings of the qualitative implementation evaluation are consistent with the quantitative effectiveness evaluation in suggesting the beginnings of favorable changes despite implementation shortcomings.

Our findings highlight the need to focus on and optimize implementation in the context of generating experimental evidence on workplace suicide prevention. There are numerous challenges to successful implementation, including the artificiality and awkwardness of randomization (for sites and workers), the reluctance of sites to grant work time for data collection, and the long timelines required for the various steps in the change process to take place. In retrospect, had we better anticipated the challenges we faced, we would have allowed at least 1 year between baseline and final assessments. This would have enabled the implementation of initial GAT training, the timely training of Connector and ASIST volunteers, the opportunity to monitor staff changes and provide top‐up GAT training to retain the 80% threshold, and adequate time for workers who experience distress with the opportunity to call upon a Connector, ASIST worker, or MATES Field Officers. Only then could distressed workers be ‘connected’ to the help they might need through interactions with site‐based MATES volunteers or Field staff, the MATES Helpline, MATES case workers, and the further help that could be facilitated by case workers [12, 21]. At the worker level, GAT training is intended to improve suicide and mental health literacy, reduce stigma, and increase help‐seeking intentions and help‐offering behaviors, setting the stage to either constructively engage with help offered or to seek help independently when in need. While there is observational evidence supporting MATES program‐associated favorable changes in these outcomes [12], there remains a need for trial‐based evidence to validate the observational.

This lack of experimental evidence is also a limitation of the broader workplace suicide prevention evaluation literature [22, 23, 24, 25]. The nature of the ultimate outcome of interest in this context: reduced suicide mortality, makes evaluation through experimental studies extremely challenging due to the need for very large studies or very long timelines to generate stable estimates of outcome between groups [26, 27]. Hence the use of intermediate outcomes in trial contexts. For example, men who have greater help‐seeking intentions as measured using the GHSQ are less likely to experience new‐onset suicidal ideation, which is presumed to flow on to lower suicide risk [28]. In contrast, large observational evaluation studies have been able to measure suicide mortality and demonstrate lower rates in intervention versus comparison groups [25, 26, 27]. Hence, even if successfully implemented workplace suicide prevention strategies show improvements in intermediate outcomes such as the GHSQ using experimental designs, they will still need to be complemented by observational studies and surveillance [29] to demonstrate reductions in suicide rates. Alternative observational evaluation approaches are also needed to complement experimental studies, including realist evaluation, natural experiments, and trial emulation approaches [30].

The MATES in Manufacturing trial was limited by the small number of sites, baseline imbalances in some key covariates, incomplete implementation, increases in the study population during the intervention period, and poor response rates at final. In addition, the planned economic evaluation [14] was precluded by very low response rates to the one‐time survey at follow‐up. Further, the scope of the MATES program is limited in not directly addressing work‐related contributions to suicide risk [27], nor did we have the resources to determine whether other programs at each site assessed and managed work‐related risks. Strengths include substantial scale of the MATES in Manufacturing trial, the real‐world conditions under which it was tested, the rigorous experimental design and analysis, and the implementation evaluation that enabled explanation of the effectiveness results. Despite the substantial implementation challenges, the study helped to address the lack of experimental studies in the area and did show signs of beginning to have the desired impacts. The main lesson from this trial, it would seem, is that complex multi‐level workplace suicide prevention (and other) interventions need long timelines with constant attention to implementation backed up by the resources required to respond to implementation shortcomings. Those resources include human (staffing), relational (e.g., organizational leadership buy‐in), administrative (e.g., allowing work time for data collection and intervention activities), and material (e.g., budget). In the context of this trial, we had a long timeline, but it was compromised by several factors, including COVID‐related and industrial delays, decline in study site organizational support over the course of the trial, and budget (the research grant provided enough to fund only a bit more than a fulltime project officer for 3 years, with all implementation costs carried by the MATES organization). Paraphrasing a recent paper in this area entitled “simple roads to failure, complex paths to success,” very few things need to fail to compromise a complex intervention, whereas nearly everything has to work to achieve success [31]. The planning of future trials should take these factors into consideration.

## 5 Conclusions

This trial of the MATES in Manufacturing intervention was not effective as implemented. This was most likely attributable to poor implementation, leaving open the question of what the impacts of the intervention would be if implemented as intended with longer follow‐up time to allow positive outcomes to develop. There remains a need for experimental evidence of effectiveness of workplace suicide prevention programs, accompanied by implementation evaluation. Greater resourcing of implementation and longer intervention periods than this trial will likely be required to demonstrate achievement of the desired outcomes. Further, we would argue for valuing strong observational evaluation evidence on a par with experimental evidence in workplace and other community settings, given the implementation challenges inherent in experimental studies.

## Author Contributions

Anthony D. LaMontagne led the study. Anthony D. LaMontagne, Christopher Lockwood, Andrew Mackinnon, Laura Cox, David Henry, and Tania L. King participated in the conception and design of the work. All authors contributed to the acquisition, analysis, or interpretation of data for the work. Anthony D. LaMontagne led the writing of the paper, and all authors contributed to revising it critically for accuracy and important intellectual content. All authors approved the final version to be published and all authors agreed to be accountable for all aspects of the work in ensuring that questions related to the accuracy or integrity of any part of the work are appropriately investigated and resolved.

## Disclosure by *AJIM* Editor of Record

Jian Li declares that he has no conflict of interest in the review and publication decision regarding this article.

## Disclaimer

The authors have nothing to report.

## Ethics Statement

The protocol was reviewed and approved by the Deakin University Human Research Ethics Committee (protocol #2021‐276).

### 1 Consent

Implied informed consent was indicated by participant written response to baseline surveys.

## Conflicts of Interest

The lead author (A.D.L.) discloses voluntary, unpaid Board membership with MATES in Construction, and having received research funding from MATES in Construction for previous projects. C.L. discloses his role as CEO of MATES in Construction. No other authors declare any conflicts of interest.

## Supporting information

Supporting information.

## Data Availability

The data that support the findings of this study are available on request from the corresponding author. The data are not publicly available due to privacy or ethical restrictions.
