# Supplementary material for: MATES in Manufacturing: A Cluster RCT Evaluation of a Workplace Suicide Prevention Program
Source: Am J Ind Med. 2025 Jan 12;68(4):331–43. doi: 10.1002/ajim.23698 (PMC11898168; doi:10.1002/ajim.23698)
Supplement: Supplementary file 1 — Supporting information. [file AJIM-68-331-s001.docx]

**SUPPORTING INFORMATION**

**MATES in Manufacturing:**

**A cluster RCT evaluation of a workplace suicide prevention program**

LaMontagne et al, 2024

**Table of Contents**

| **Topic** | **Page** |
| --- | --- |
| Primary outcome analyses   - *GHSQ by Gender* - *GHSQ by Occupational Status* - *Participants who were K6 cases at baseline* - *Baseline GHSQ Conditional analysis* | 2  2  2  3 |

GHSQ PRIMARY OUTCOME ADDITIONAL ANALYSES

GHSQ Help-seeking intentions

Following the protocol, outcomes by gender and occupational sub-groups were explored.

*GHSQ Help-seeking intentions by Gender*

Subgroup analyses were undertaken for males and females. There was a (just) significant differential effect for males (p=0.0499). However, as can be seen in Figure 3, the mean GHSQ score in the intervention group was slightly lower than in the control group at baseline and slightly higher post intervention so that the endpoint effect size was very small.

The reverse pattern was seen in the female analysis, but with much larger between occasion and between group differences. Differential change was significant and, despite a slightly higher baseline mean, the intervention group had a significantly lower post intervention mean than the control group (difference: 5.84, t=2.12, df=183.5, p=0.0353). The small to moderate effect size of 0.39 was not significant (see Table 1 below); wide confidence intervals reflect the small number of women in the study.

*GHSQ Help-seeking intentions by Occupational Status*

Separate analyses were undertaken for workers classified as white or blue collar workers. There was no evidence of any differential effect in white collar workers (see Table 1 and Figure 4). The baseline advantage in the intervention group was maintain post intervention but the between group difference was not significant on either occasion nor was the change in either group over time.

Although the pattern of means for blue collar workers was less regular, the conclusion of no differential change also applied. At baseline, mean GHSQ in the intervention group was lower than in controls, with this reversing post intervention. However, differential change was not significant (p=0.1172) and neither between group was not significant on either occasion nor change in either group over time was significant.

In addition, exploratory analyses were conducted to determine if participants who were K-6 ‘cases’ at baseline were more beneficially affected by the intervention that non-cases.

*Participants who were K6 cases at baseline*

As this was a universal intervention individuals not experiencing psychological distress may show little change in the outcomes assessed. Increases in potential help sources might be seen only in participants with elevated distress at entry to the trial. The sample included few participants with scores of 13 or above, so a lower threshold of 5 or above was used to define cases of moderate distress (see Prochaska et al., 2012).

Somewhat surprisingly, preliminary analysis showed that the baseline mean GHSQ score for cases was 5.94 points lower than non-cases. This was statistically significant (t=4.53, df=1098, p<0.0001) although the difference was somewhat lower in the intervention group (2.92 versus 5.94).

Despite having greater headroom to increase, there was almost no differential effect on GHSQ scores attributable to the intervention in this subgroup of individuals with psychological distress, and no statistically significant differences were found (see Table 1).

*Baseline GHSQ Conditional analysis*

Figure 6 shows a scattergram of post intervention scores against baseline values. Simple linear regressions and loess curves fitted separately to each group are also shown. Whereas the MMRM models focus on group averages (the margins), conditional models focus on the predicted response of individuals conditional their baseline status. This view and an associated analysis of covariance can help identify group differences that differ as a function of baseline GHSQ status: it may be that participants with already-high scores show no benefit from the intervention while those with initially low scores may have more ‘headroom’ to do so.

In this case, regression lines for each group are parallel and very close across the range of baseline scores. This indicates likely absence of any baseline-dependent effect and the closeness of the lines reflects the absence of any effect at any level. This is consistent with the outcome of the completers analysis.

This was formalised in mixed effects ANCOVA models which included a random site intercept to account for clustering. The basic model included baseline GHSQ score and group assignment as predictors. This implies that any effect of the invention is constant over the range of baseline GHSQ scores. An elaboration of this model adds an interaction between group and baseline score which allows for an incremental effect of group as a function of baseline score. None of these effects were significant and contrasts demonstrated the non-significance of group differences across the full range of baseline scores.

*Summary*

Except for gender subgroup analyses, these further analyses failed to demonstrate any effect of the intervention on GHSQ scores. No effects were found in participants with moderate or greater distress as defined by a baseline K6 score of 5 or higher. Surprisingly, this subgroup had lower baseline scores on the GHSQ.

Significant differential effects of the intervention were found in opposite directions for males and females, the latter being substantially larger and more robustly significant. Any interpretation of these effects must be tempered by non-significant baseline differences which amplified change from baseline to post intervention. Nevertheless, the potential important of gender on outcomes must be considered – contrary effects in males and females could wash out the effects in whole-sample analyses.

*Table 1* GHSQ results for outcomes post intervention

| Outcome /Group | Difference in Change^1^ | P value | Effect Size |
| --- | --- | --- | --- |
| ITT | 1.52  (-0.69 – 3.74) | 0.1767 | 0.06  (-0.11.– 0.26) |
| Completers | 1.38  (-1.50.– 4.26) | 0.3452 | 0.06  (-0.21.– 0.32) |
| Gender - Males | 2.39  (0.001– 4.79) | 0.0499 | 0.08  (-0.11 – 0.27) |
| - Females | -8.40  (-14.90 – -1.89) | 0.0118 | -0.39  (-1.33 – 0.54) |
| Status - White collar | -0.06  (-3.99 – 3.88) | 0.9775 | 0.17  (-0.12 – 0.45) |
| - Blue collar | 2.39  (-0.56 – 5.34) | 0.1127 | 0.06  (-0.19 – 0.30) |
| K6 baseline cases | 1.09.  (-2.24–4.41) | 0.5209 | 0.11  (-0.09 – 0.32) |

1 Change from baseline to endpoint for intervention compared to control group


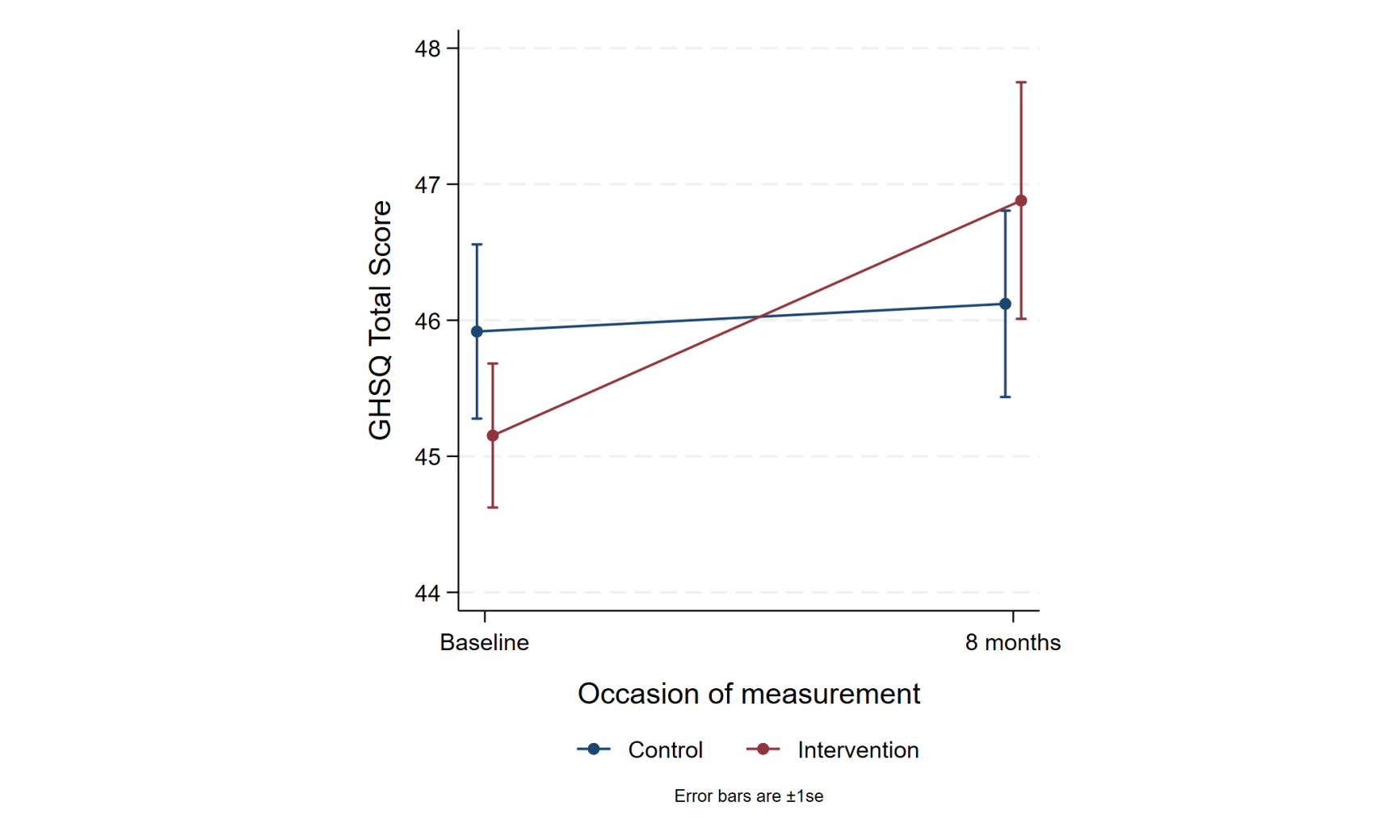


*Figure 1* Estimated marginal means for each intervention group on each occasion of measurement


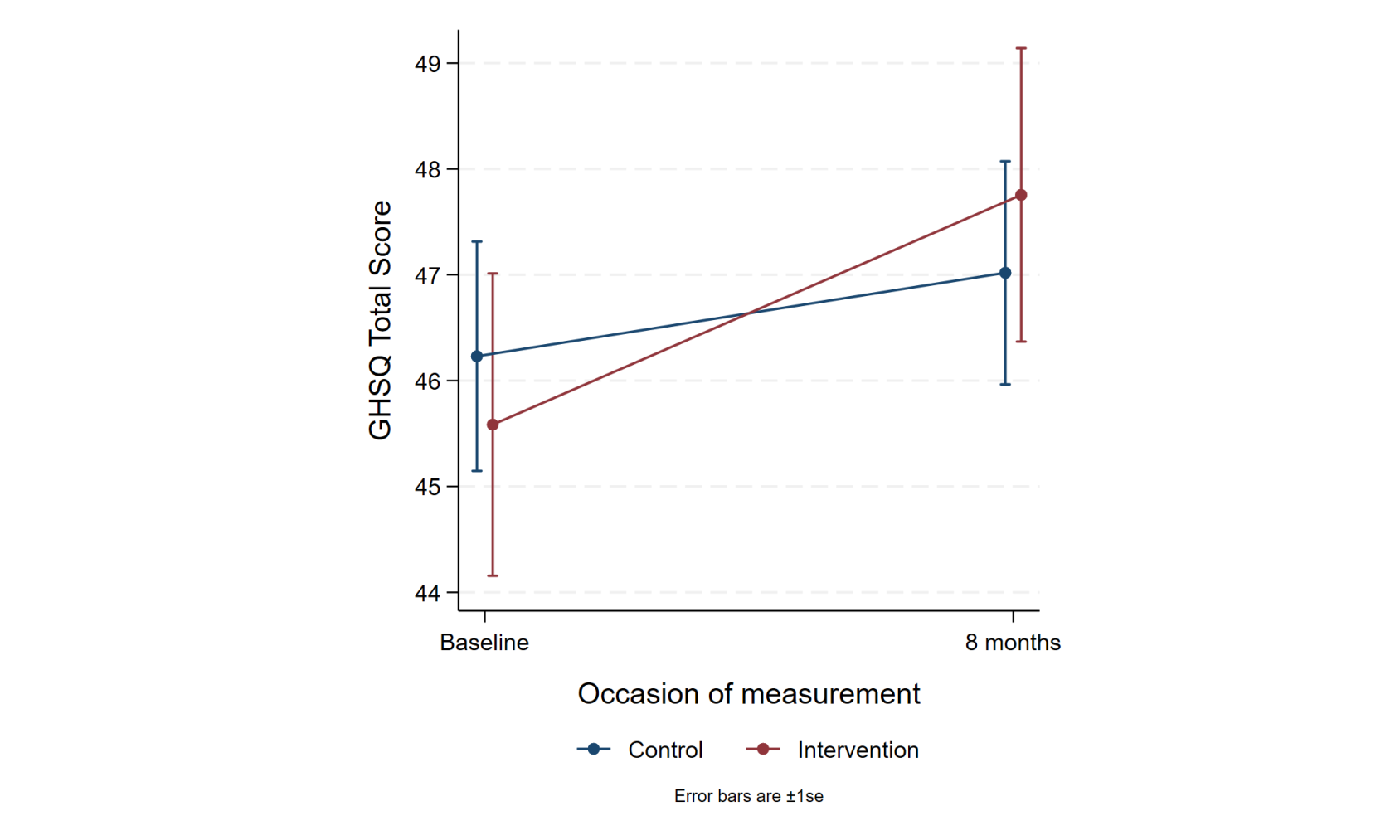


*Figure 2* Estimated marginal means for each intervention group on each occasion of measurement in participants with observations at both baseline and post intervention.


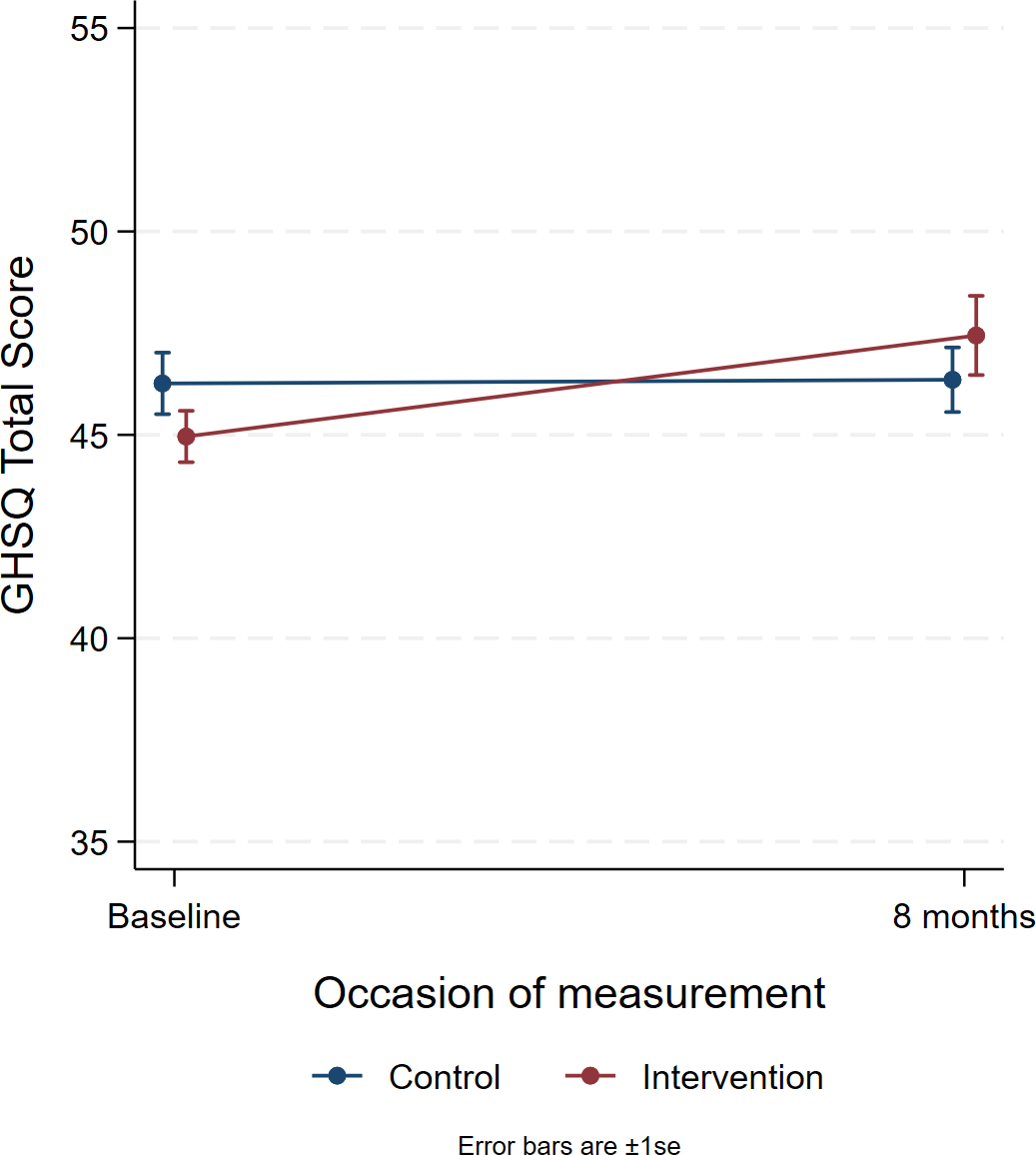

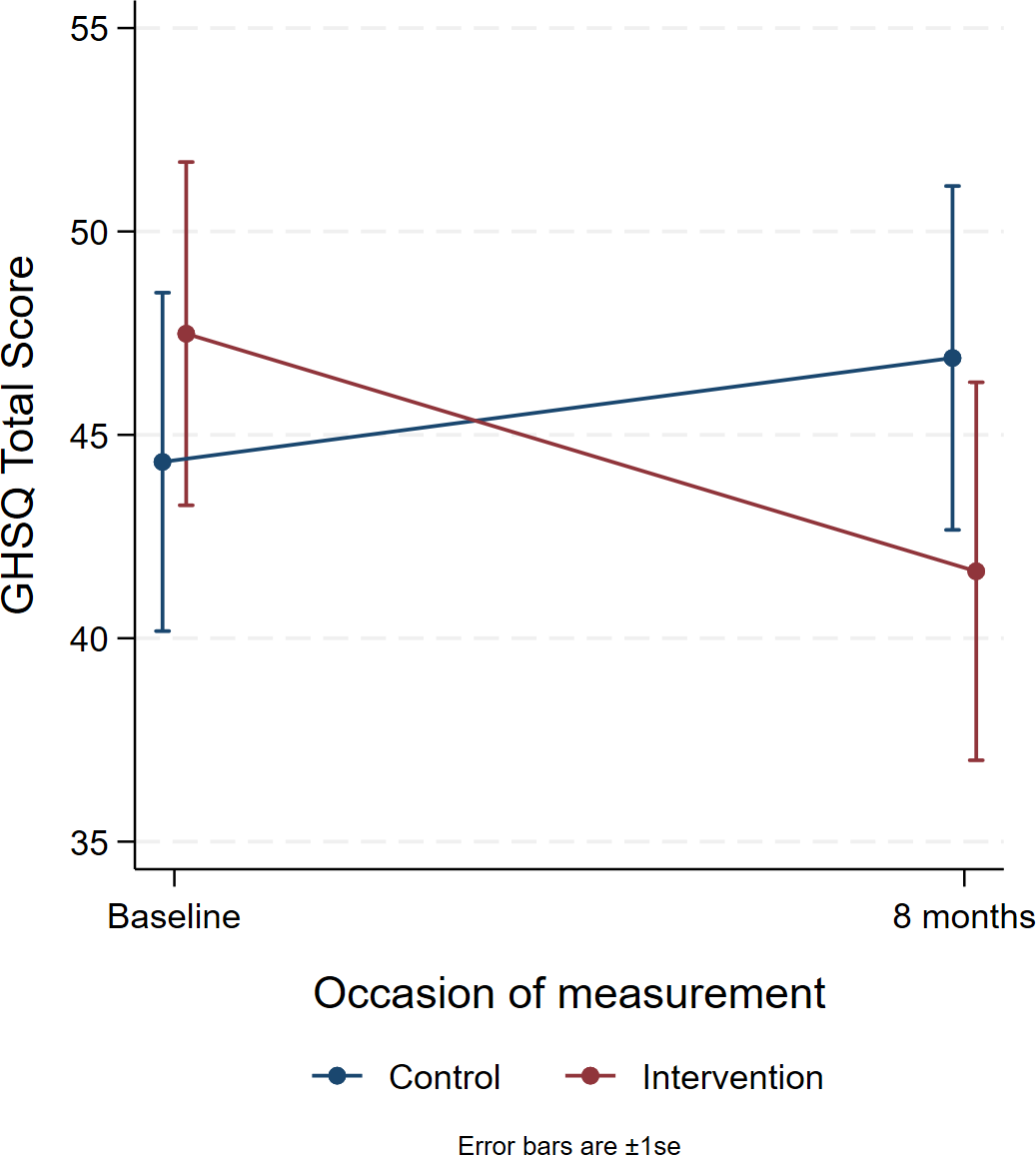


*Figure 3* Estimated marginal GHSQ means for males (left) and females (right).


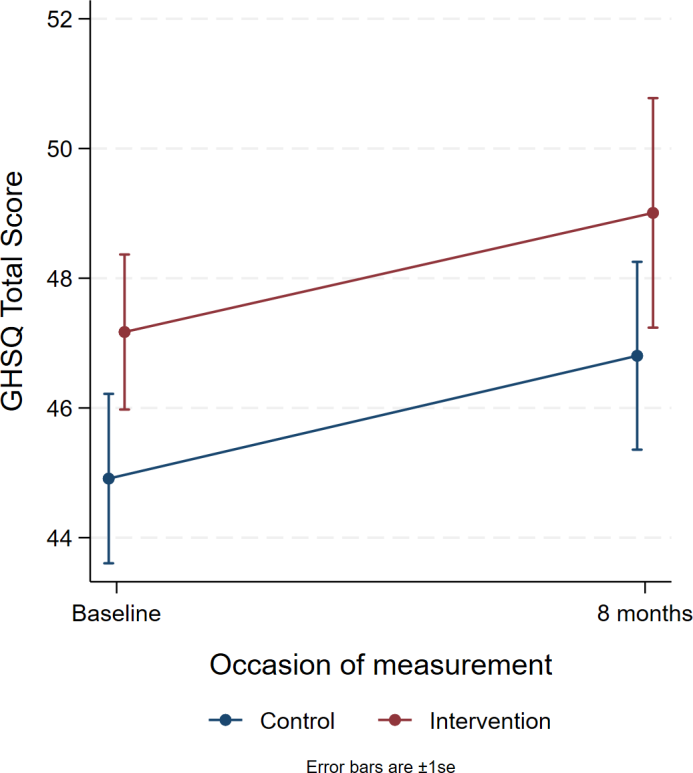

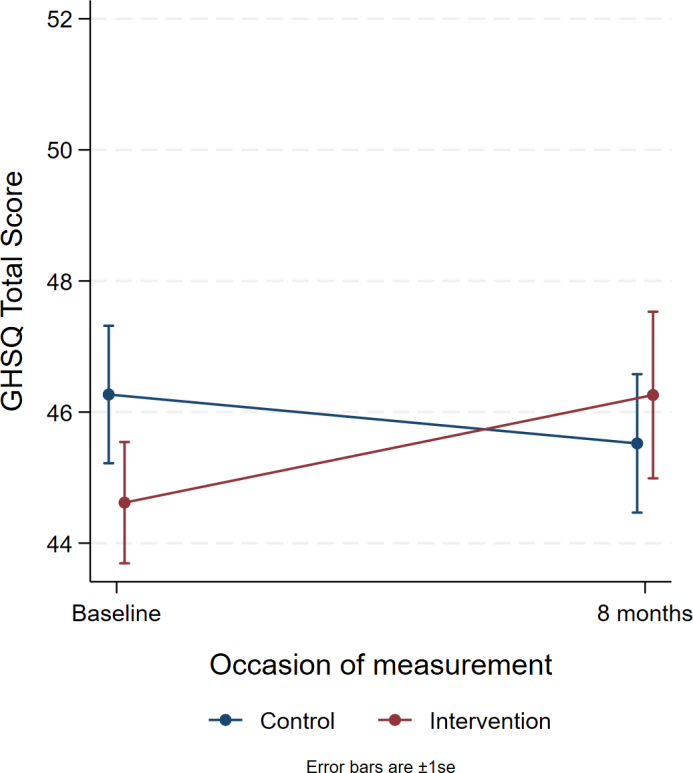


*Figure 4* Estimated marginal GHSQ means for white (left) and blue (right) collar workers.


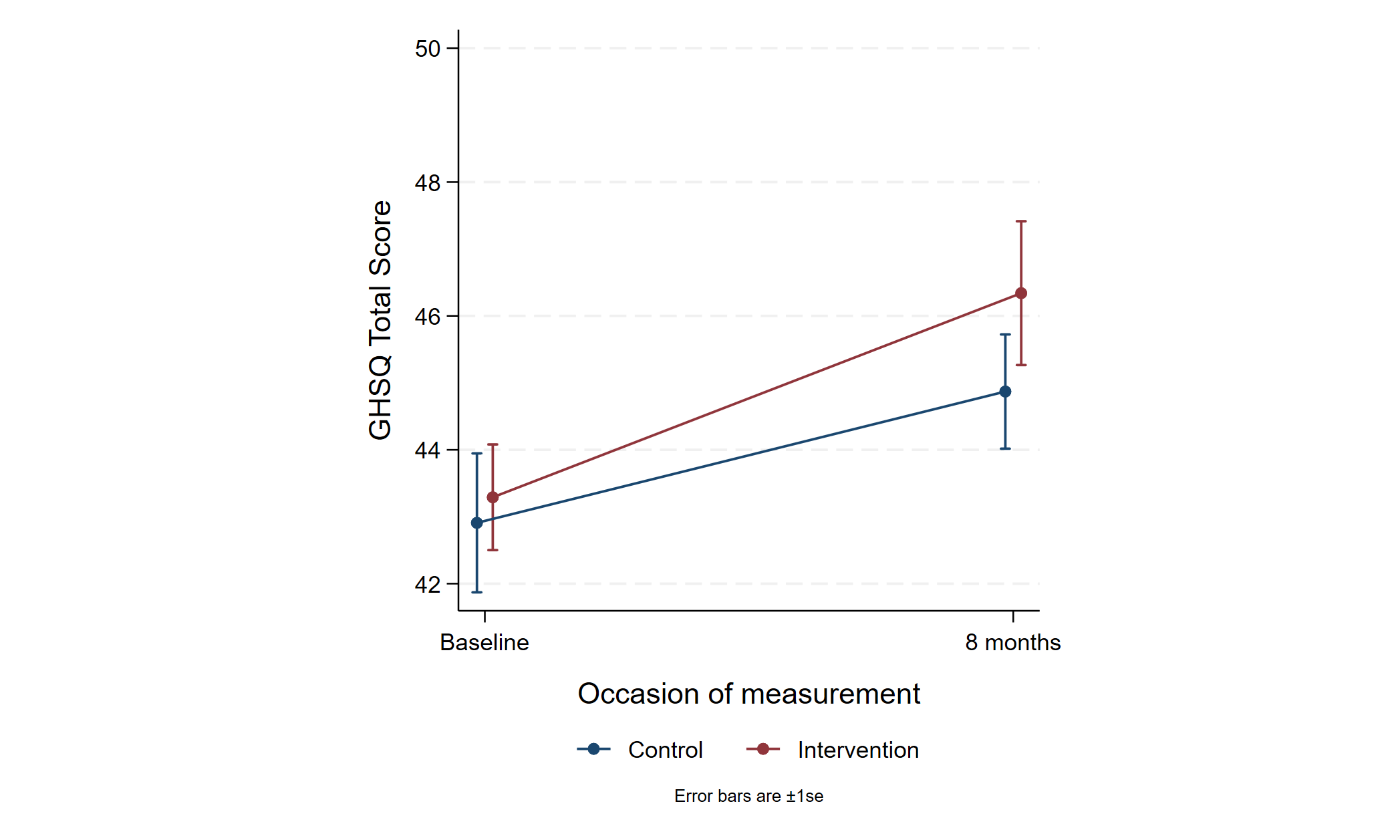


*Figure 5* Estimated marginal GHSQ means for each intervention group on each occasion of measurement in participants with a baseline K6 score of 5 or above.


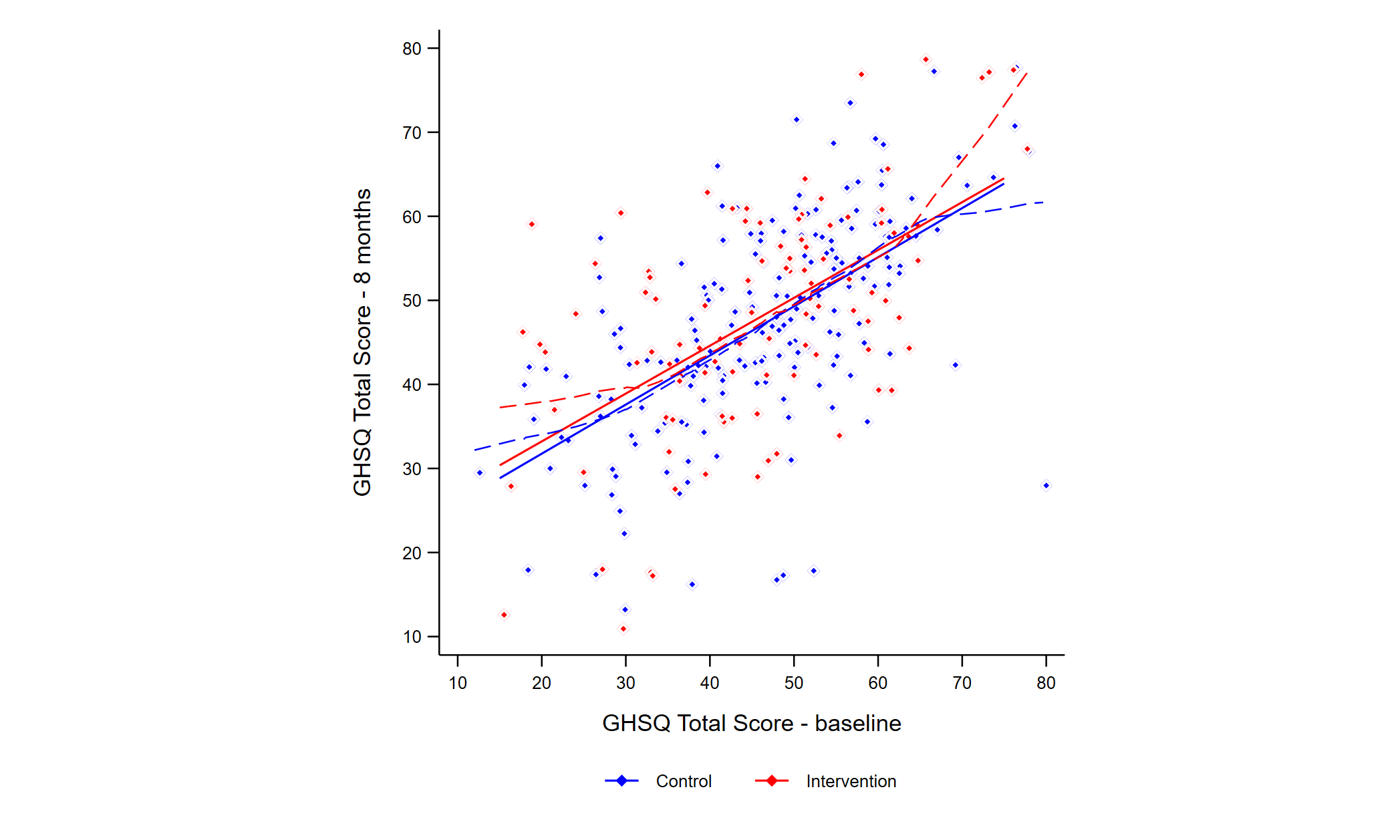


*Figure 6* Scattergram of post intervention versus baseline GHSQ scores with regression lines and loess curves for each group.

*Reference*

Prochaska, J.J., Sung, H.-Y., Max, W., Shi, Y. and Ong, M. (2012), Validity study of the GHSQ scale as a measure of moderate mental distress based on mental health treatment need and utilization. *Int. J. Methods Psychiatr. Res*., **21**: 88-97. <https://doi.org/10.1002/mpr.1349>
